# A 15-Year-Old Boy in Long-Term Remission of Epileptic Seizures With Infantile-Onset Attenuated Nonketotic Hyperglycinemia

**DOI:** 10.7759/cureus.98474

**Published:** 2025-12-04

**Authors:** Tomoyo Itonaga, Osamu Kobayashi, Kenji Ihara

**Affiliations:** 1 Department of Pediatrics, Faculty of Medicine, Oita University, Oita, JPN; 2 Department of Pediatrics, School of Medicine, Oita University, Oita, JPN

**Keywords:** acute encephalopathy, amt gene, febrile illness, infantile spasms, long-term survival, nonketotic hyperglycinemia, seizure remission, sodium benzoate

## Abstract

Nonketotic hyperglycinemia (NKH) is a rare autosomal recessive disorder caused by defects in the glycine cleavage system. Most patients with early-onset NKH present with severe manifestations, including poor neurological outcomes and refractory seizures. However, a proportion of neonatally presenting patients exhibit the attenuated phenotype, characterized by variable clinical features. We report a boy with infantile-onset NKH who achieved long-term seizure remission into adolescence. A three-month-old boy developed infantile spasms refractory to standard antiepileptic drugs and worsened by valproic acid. Laboratory findings revealed elevated glycine levels in plasma and cerebrospinal fluid with an increased CSF/plasma ratio of glycine. A glycine breath test indicated partial reduction of glycine metabolism. He was diagnosed with early-onset NKH at nine months, and thereafter, specific treatments were started with sodium benzoate and dextromethorphan. He exhibited severe developmental delay under sustained seizure control. Genetic testing at 11 years of age revealed compound heterozygous variants in the *AMT* gene: a novel missense variant (p.L116R) and a pathogenic frameshift variant (p.P20fs*76). At 15 years, he presented with tremor as rhythmic limb shaking and lethargy with abnormal 5 Hz waves in the electroencephalogram during influenza A, whereas any epileptic seizures did not occur. No suggestive findings of encephalitis were observed on brain MRI or CSF analysis, but steroid pulse therapy was administered along with baseline NKH therapy. No seizures occurred, and neurological findings seemed unaffected by this episode. This case illustrates that attenuated NKH can be well managed with sustained seizure-free status into adolescence, even in and after subclinical influenza encephalopathy, suggesting potential prophylactic effects of sodium benzoate and dextromethorphan.

## Introduction

Nonketotic hyperglycinemia (NKH) is a rare inborn error of glycine metabolism caused by defects in the glycine cleavage system, comprising four protein complexes (P, T, H, and L), encoded by *GLDC, AMT, GCSH*, and *DLD* genes, respectively [1,2]. Genotype-phenotype correlations have been reported and broadly associated with clinical symptoms and long-term outcomes, particularly in relation to residual activity of the complex with *AMT* (T-protein) variants [2-4].

The clinical manifestations are classified as severe or attenuated types [5,6]. Patients at neonatal onset within days of birth typically demonstrate lethargy, hypotonia, apnea, hiccups, and intractable seizures, often leading to profound psychomotor disability, classified as a severe type. Many of those die during the neonatal period, and the mortality rate of survivors in infancy is reported to be high [7,8]. In contrast, attenuated type NKH presents with more variable outcomes, and some patients achieve walking, speech, and even relatively normal cognitive function. Up to 15% of neonatally presenting patients and 50% of infantile-onset patients are classified as having the attenuated type [8].

No standardized protocol for NKH treatment has been established, and management is primarily supportive to reduce glycine accumulation and mitigate its neurotoxic effects on the central nervous system. Sodium benzoate and N-methyl-D-aspartate (NMDA) receptor antagonists have been applied for both types of NKH. Sodium benzoate can decrease blood glycine levels by conjugation of glycine into hippurate, thereby enhancing renal excretion of glycine. NMDA receptor antagonists, such as dextromethorphan or ketamine, are applied to block excitatory neurotransmission induced by excessive glycine [9,10]. Favorable long-term neurodevelopmental outcomes have been reported for the NKH attenuated type, whereas the neuroprotective or antiepileptic effects remained unclear in high-glycine environments or under hypercytokine conditions induced by viral infections.

We present the clinical course of a patient with infantile-onset NKH who achieved seizure remission for over 15 years under treatment with antiepileptic drugs, sodium benzoate, and dextromethorphan, notably without experiencing seizures during and after an episode of influenza encephalopathy.

## Case presentation

**Figure 1:**
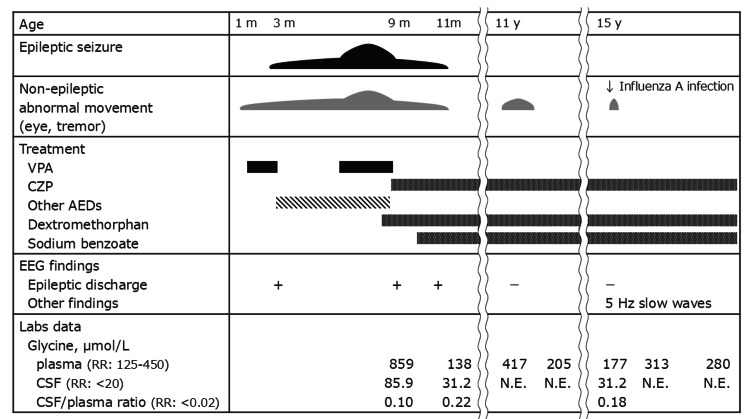
Clinical course. The figure summarizes epileptic seizures, non-epileptic abnormal movements, treatment with antiepileptic drugs, sodium benzoate, and dextromethorphan, EEG findings, and plasma/cerebrospinal fluid glycine levels from infancy to adolescence. Abbreviations: m, month(s); y, year(s); VPA, valproic acid; CZP, clonazepam; AEDs, antiepileptic drugs; CSF, cerebrospinal fluid; RR, reference range; N.E., not examined.

A boy was born at 35 weeks and four days of gestation by emergency cesarean section due to non-reassuring fetal status with neonatal asphyxia. He showed lethargy, bradycardia, and generalized hypotonia in the neonatal period. At around one month of age, occasional upward eye deviation and head-nodding were noted, and at three months, he developed frequent myoclonic jerks and generalized clonic seizures. Electroencephalography demonstrated hypsarrhythmia predominantly in the right hemisphere. Brain MRI revealed hypoplasia of the corpus callosum and delayed myelination for his age [11]. Seizures were refractory to standard antiepileptic drugs and appeared to be exacerbated by valproic acid, which also induced complex partial seizures accompanied by oral automatisms and pedaling behaviors. Biochemical testing showed elevated plasma glycine (859 µmol/L; reference range (RR) = 125-450), and cerebrospinal fluid glycine (85.9 µmol/L; RR <20), with a CSF/plasma ratio of 0.10 (RR <0.02), consistent with attenuated NKH. The [1-^13^C] glycine breath test showed a partially reduced recovery rate of 18.0% (-1.5 SD). At nine months, he was diagnosed with NKH, and treatment with sodium benzoate, dextromethorphan, and clonazepam led to complete seizure cessation. Clonazepam was continued as maintenance therapy (Figure 1).

His infantile clinical course was previously reported by Itonaga et al. [11]. At the age of two years, he presented with severe developmental delay, being able to sit alone and take oral feeding but requiring continuous assistance in daily activities and not achieving higher milestones such as walking or speech. He developed tremors during febrile illnesses, but no clinical signs indicative of epileptic seizures were observed. At 11 years, he developed abnormal eye movements during episodes of elevated plasma glycine levels without epileptic discharges in electroencephalography. These symptoms resolved after increasing the dose of sodium benzoate. At this time, the diagnosis of NKH by *AMT* gene variants was genetically confirmed, with compound heterozygous variants in the *AMT* gene; one was a novel missense variant (c.347T>G; p.Leu116Arg), at the same residue (p.Leu116Pro) already reported as pathogenic [2], and the other was a previously reported pathogenic frameshift variant (c.59del; p.Pro20ArgfsTer76) [12].

**Figure 2:**
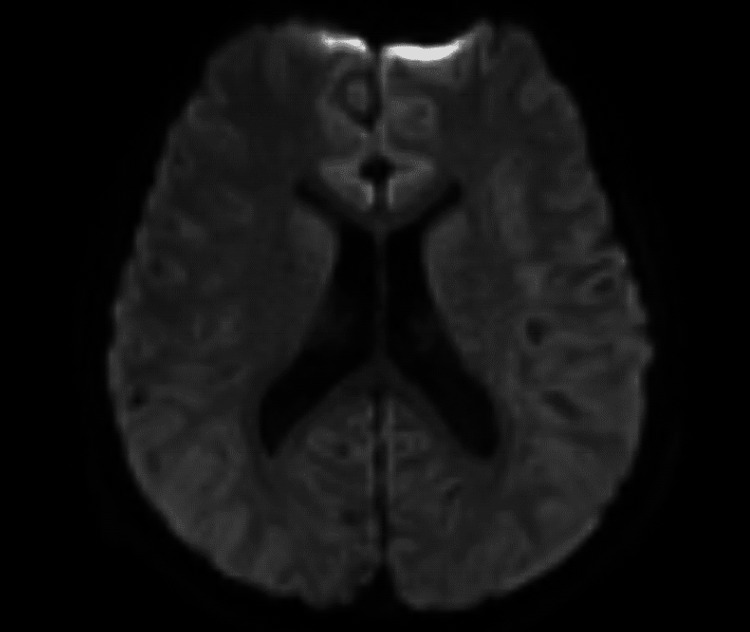
Brain MRI (axial diffusion-weighted images). There were no abnormal lesions.

**Figure 3:**
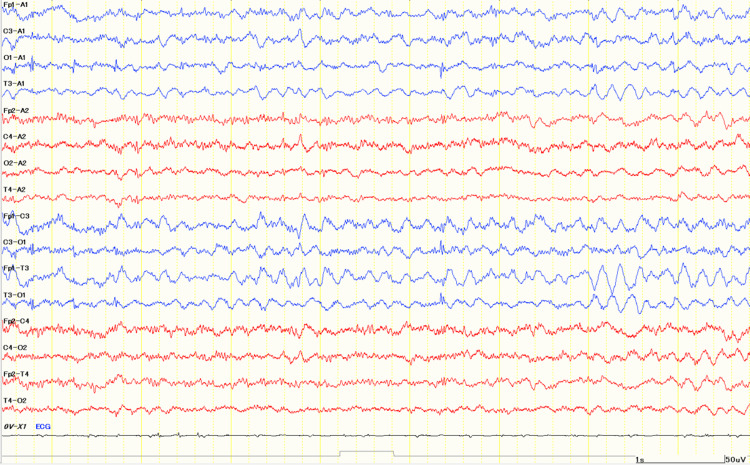
EEG on day five. The electroencephalogram on day five demonstrated 5 Hz slow waves. Electrode abbreviations: Fp1, left frontopolar; Fp2, right frontopolar; F3, left frontal; F4, right frontal; C3, left central; C4, right central; T3, left temporal; T4, right temporal; P3, left parietal; P4, right parietal; O1, left occipital; O2, right occipital; A1, left earlobe (reference); A2, right earlobe (reference).

At the age of 15 years, he was admitted to our hospital on day five of influenza A infection with pneumonia. Bacterial culture and multiplex viral screening of CSF were both negative. Lethargy under high-grade fever (38.9°C), and rhythmic 2-3 Hz myoclonus was observed without features of epileptic seizures under the neurological state of Glasgow Coma Scale E4V1M4. Laboratory data showed thrombocytopenia, elevated creatine kinase, high glycine levels in plasma (177 µmol/L; RR = 125-450) and cerebrospinal fluid (31.2 µmol/L; RR <20), with a CSF/plasma ratio of 0.18 (RR <0.02). Glycine levels were lower than those observed in infancy prior to the initiation of treatment with sodium benzoate or dextromethorphan. Brain MRI showed no abnormal findings, suggesting encephalopathy or encephalitis, such as high signal intensity on diffusion-weighted images (Figure 2), whereas the electroencephalogram demonstrated continuous abnormal 5 Hz slow waves, suggestive of encephalopathy (Figure 3).

Medical management included three-day intravenous methylprednisolone pulse therapy with baseline NKH therapy (sodium benzoate, dextromethorphan, and clonazepam) because the neurological manifestations might be caused by either a cytokine storm-type encephalopathy or an acute exacerbation of NKH. By day five of hospitalization, consciousness and myoclonus had improved, and the 5 Hz slow waves on EEG had resolved. He was discharged without additional neurological deficits. He is now 15 years of age and has remained seizure-free for more than 15 years under the treatment of antiepileptics with sodium benzoate and dextromethorphan.

## Discussion

This case report presented a clinical course of infantile-onset attenuated NKH with long-term survival and seizure remission for more than 15 years. He experienced an episode suggestive of acute encephalopathy during influenza A infection at 15 years, and recovered without apparent seizures or additional neurological sequelae.

Postnatal survival in neonatal-onset NKH has been reported as ranging from 2.3 months to 15 years. Details of the clinical course have not been well-documented, and survival beyond 10 years has been reported in only three patients with neonatal- or infantile-onset NKH (Table 1) [6,13,14].

**Table 1:** List of neonatal or infantile-onset NKH followed-up for ≥10 years of age. * Our case. M, male; F, female; m, month(s); d, day(s); y, year(s); SB, sodium benzoate; Dex, dextromethorphan, N.D., not described; DQ, developmental quotient.

No.	Sex	onset	Gene variant	Treatment			Last visit			Ref.
				SB	Dex	Others	Age	Seizure	Development	
1	F	1 d	GLDC	+	+	N.D.	11 y	Remission from 4 y	Independent walk at age 5 y (DQ 37)	[8]
2	M	3 m	AMT	+	+	Antiepileptic drug	15 y	Remission	Severe delay (sit alone)	*
3	M	2 d	GLDC	+	+	Multiple antiepileptic drugs	17 y	Refractory	Severe delay (bedridden)	[13]
4	F	4 m	N.D.	+	+	Low glycine diet	18 y	Never	N.D.	[14]

All four patients, including the present case, received the specific drugs, sodium benzoate and dextromethorphan. One of the neonatal-onset cases exhibited refractory seizures under treatment with multiple antiepileptic drugs, along with severe developmental delay. In contrast, the other three neonatal-onset survivors had no recurrence of seizures since the age of four years, with or without treatment. Overall, seizure severity considerably varied, ranging from refractory epilepsy to long-term seizure remission. Neurodevelopmental improvement is anticipated in patients with the attenuated form of NKH when combination therapy is initiated in the early neonatal period, and glycine levels are successfully normalized. In NKH, elevated glycine enhances NMDA receptor activation, lowering the seizure threshold. This mechanism provides a rationale for a dual therapeutic approach: reducing glycine concentrations with sodium benzoate and inhibiting NMDA receptor activity with dextromethorphan. Although previous reports have suggested that the combination of benzoate and dextromethorphan may be effective for seizure control, glycine normalization varies markedly among patients. Dextromethorphan may be more advantageous in the long-term management of attenuated NKH than in the severe phenotype [5]. In our patient, febrile illnesses caused by influenza may increase catabolism, resulting in elevated endogenous glycine production and theoretically lowering the seizure threshold [15-20]. In addition, cytokine storm could synergistically increase the risk of convulsion. Even under these conditions, he did not experience seizures, suggesting the prophylactic antiepileptic effect of sodium benzoate or dextromethorphan. This is an isolated case, and definitive conclusions cannot be drawn; therefore, further accumulation of similar cases is required to determine the potential preventive effects of sodium benzoate and dextromethorphan for NKH.

## Conclusions

This case highlights the potential prophylactic effect of sodium benzoate and dextromethorphan in attenuated NKH, supporting long-term seizure remission even during influenza infection.
